# Sutureless Dehydrated Amniotic Membrane (Omnigen) Application Using a Specialised Bandage Contact Lens (OmniLenz) for the Treatment of Dry Eye Disease: A 6-Month Randomised Control Trial

**DOI:** 10.3390/medicina60060985

**Published:** 2024-06-15

**Authors:** Sònia Travé-Huarte, James S. Wolffsohn

**Affiliations:** College of Health and Life Sciences, Aston University, Birmingham B4 7ET, UK; j.s.w.wolffsohn@aston.ac.uk

**Keywords:** dry eye disease, dehydrated amniotic membrane, randomised controlled trial, ocular surface disease index, corneal nerve regeneration

## Abstract

*Background and Objectives*: Dry Eye Disease (DED) is a chronic condition characterised by tear film instability and ocular surface disruption, significantly impacting patients’ quality of life. This study aimed to provide top-level clinical evidence for the long-term efficacy of dehydrated amniotic membrane (dAM, Omnigen^®^) delivered via a specialised bandage contact lens (sBCL, OmniLenz) for managing moderate-to-severe DED. *Materials and Methods*: This randomised controlled trial (NCT04553432) involved 93 participants with moderate-to-severe DED, randomised to receive a 1-week bilateral treatment of either dAM (17 mm diameter with 6 mm central ‘window’) applied under a sBCL or sBCL alone. Participants were assessed at baseline and followed up at 1, 3, and 6 months post-treatment. Outcomes included changes in symptomatology, tear film and ocular surface measurements, and in vivo confocal microscopy imaging of corneal nerve parameters and corneal dendritic cell (CDC) counts. *Results*: The dAM-sBCL group demonstrated a 65% reduction in OSDI scores at 6 months (*p* < 0.001), with 88% of participants showing improvement at 1 month. Corneal staining was significantly reduced in both groups. dAM-sBCL provided significant improvements in corneal nerve parameters at 1 month, with sustained positive trends at 3 months. Additionally, dAM-sBCL significantly reduced mature CDC counts, suggesting an anti-inflammatory effect. *Conclusions*: Treatment with dAM-sBCL for just 1 week significantly and rapidly improved dry eye symptoms as well as ocular surface signs for at least 3 months. It also enhanced corneal nerve health while reducing activated/mature corneal inflammatory cell numbers, presenting a safe and promising new treatment for moderate-to-severe DED.

## 1. Introduction

Dry Eye Disease (DED) is a multifactorial ocular disorder characterised by tear film instability and disruption of ocular surface homeostasis, leading to potentially severe discomfort [1]. The prevalence of DED, estimated using the standardised Tear Film and Ocular Surface Society (TFOS) diagnostic criteria [2], ranges from 18 to 58% [3,4,5,6]. Due to the chronic nature of the condition, DED has seen a notable increase in prevalence, approximately tripling between 2005 and 2012 [7]. The pathogenesis of DED involves inflammation and damage to the ocular surface [8,9], with tear film instability contributing to increased mechanical stress between the eyelids and the ocular surface, leading to epitheliopathy and heightened patient symptomatology. Progressive deterioration of corneal nerves and an increase in corneal dendritic cells (CDCs) may play a pivotal role in disease progression, exacerbating symptoms and ocular surface damage through disrupted inflammatory and nociceptive pathways [10]. CDCs play a pivotal role in the ocular surface immune response, serving as antigen-presenting cells that can modulate inflammation. In the context of DED, the activation state of CDCs, categorised as either immature (non-activated) or mature (activated), can significantly influence disease progression and symptom severity [11].

The detrimental impact of DED on quality of life can be profound [12], comparable to living with conditions such as moderate angina or a disabling hip fracture [13]. DED is the sixth most common reason for seeking ocular medical services, with an annual incidence rate of approximately 0.9%, surpassed only by refraction/accommodation disorders and cataracts [14]. Approximately 9.2 million Americans [15] and 5.2 million British individuals [16] suffer from moderate-to-severe DED. Economically, DED places a significant burden on healthcare systems, with costs approximating USD 3.84 billion annually in the United States and an overall societal cost of around USD 55.4 billion [14,17].

Considering these challenges, the 2017 TFOS second dry eye workshop report (TFOS DEWS II) outlined a stepwise treatment approach for DED management [18], which includes medications such as artificial tears, ocular lubricants, and non-glucocorticoid immunomodulatory and LFA-1 antagonist drugs as frontline treatments. In 2014, England’s National Health Service (NHS) incurred over £27 million in expenses from 6.4 million prescriptions for these treatments alone. However, further therapeutic options include punctal plugs (primarily for aqueous tear deficiency), warm compresses, lid hygiene, and intense pulse light therapy (primarily for meibomian gland dysfunction) [19], as well as therapeutic bandage contact lenses and serum eye drops, which can be prescribed depending on the disease’s severity and underlying cause [18,20]. Despite the plethora of available treatments, the absence of comparative safety or efficacy evidence, complicates optimal treatment selection [21].

Amniotic membrane transplantation has demonstrated anti-inflammatory, anti-angiogenic, and potent healing properties in ocular surface disease treatment [22,23,24,25,26,27], making it a promising option for DED treatment. Although traditionally accessible only through specialised surgical ophthalmology pathways, making it an end-of-line treatment option for severe DED [18], recent innovations in sutureless amniotic membrane transplantation have broadened its availability into a non-surgical, outpatient ‘in-office’ setting. However, challenges such as cold chain logistics, the requirement for maintained storage conditions, and patient tolerance have historically remained [28], limiting its use in routine DED management.

In a novel development, sutureless application of human amniotic membrane-derived dry matrix (Omnigen^®^) using a specialised therapeutic bandage contact lens (OmniLenz^®^, both NuVision Biotherapies, Nottingham, UK) has shown promise in treating persistent epithelial defects [29,30] and acute chemical burns [31,32], demonstrating a safe and effective treatment with a well-tolerated length of wear.

Our preliminary research indicated that a one-week bilateral treatment with sutureless Omnigen significantly ameliorated symptoms in DED patients with moderate-to-severe disease for at least one month [33]. Expanding on this foundation, this randomised controlled trial evaluated the prolonged efficacy of this novel bilaterally applied sutureless human amniotic membrane-derived dry matrix (Omnigen) treatment in mitigating both signs and symptoms of moderate-to-severe DED over six months. This study also assessed the impact of the treatment on corneal nerve health and presumed inflammatory cell dynamics, which are critical to understanding the comprehensive effects of our intervention on ocular surface pathology in DED management.

## 2. Materials and Methods

**Figure 1:**
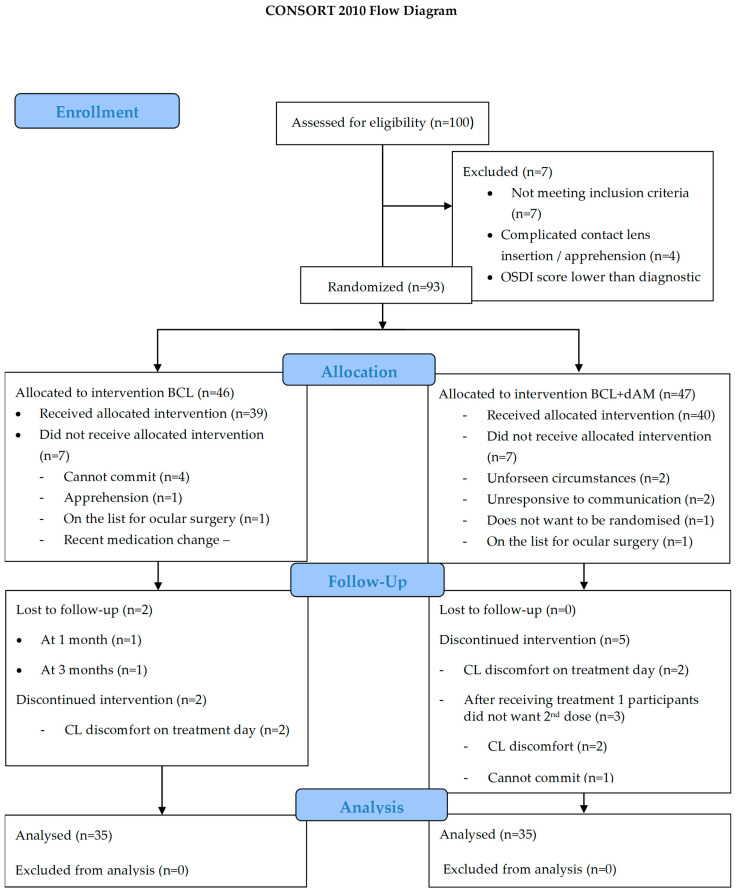
Consolidated standards of reporting trials 2010 flow design (CONSORT).

This prospective, double-masked, randomised controlled trial was registered on ClinicalTrials.gov (NCT04553432), adhered to the CONSORT statement (Figure 1) [34], followed the tenets of the Declaration of Helsinki, and received approval by the Aston University Research Ethics Committee (#1612). Written informed consent was obtained from all participants before entering the study.

### 2.1. Participant Selection

Participants were required to be ≥18 years old with a longstanding (at least 1-year) positive diagnosis of moderate-to-severe DED, as defined by an Ocular Surface Disease Index (OSDI) score of between 25 and 80, and refractory to artificial tears and lid management. Clinical inclusion criteria included at least one of the following clinical signs: corneal (≥5 punctate spots) or conjunctival (≥9 punctate spots) staining, or a non-invasive Keratograph breakup time (NIKBUT) ≤8 s. Participants had no changes in DED therapy in the 6 weeks prior to their baseline visit. Exclusion criteria included a history of ocular herpetic keratitis, recent (within ≤6 months prior to the baseline visit) ocular surgery or Intense Pulsed Light therapy (IPL), current use of glaucoma or other medications known to alter the tear film [35], use of moisture chamber goggles, active ocular surface pathologies other than DED, eyelid abnormalities or extensive ocular scarring, and allergies to specific antibiotics or antimycotics (gentamycin, imipenem, nystatin, polymyxin B, and vancomycin).

### 2.2. Study Design

Patients attending for the treatment of DED were assessed for suitability on an ongoing basis. The study involved seven appointments: enrolment (day −30), baseline/treatment (day 0), treatment replacement (days 4–5), treatment removal (days 8–10), and post-treatment follow-ups at one month (day 30), three months (day 90), and six months (day 180). The six-month follow-up involved the completion of a paper-based symptomatology and quality of life questionnaire. Eyes were treated bilaterally, but data for ocular surface homeostasis markers was analysed from the right eye only. The required sample size for the calculated study power was 33 participants per group (G*Power v3.1) based on the primary outcome detecting a minimal clinically important difference (MCID) of 4.5 to 7.3 in the OSDI [36] score with 95% power (β = 0.05) at a two-sided statistical significance level of 5% (α = 0.05). More participants were screened and randomised to allow for drop-out rates.

Participants were randomly assigned using computer-generated random numbers as they enrolled sequentially. The randomisation schedule was established before participant recruitment, ensuring that the investigator conducting the initial assessments had no role in assigning treatments. Participants were randomised into two groups, both receiving two courses of treatment: either Omnigen + OmniLenz combination (dAM-sBCL group) (*n* = 35) or OmniLenz BCL-only (sBCL-only group) (*n* = 35). To avoid different symptomatology scores between eyes, both eyes were treated, but data was collected only for the right eye.

### 2.3. Intervention

All participants received two consecutive 4–5-day bilateral applications of their respective treatment; the dAM-sBCL group received Omnigen dAM (17mm diameter disc with a 6 mm central aperture) applied under the OmniLenz specialised soft bandage contact lens (sBCL, 18 mm diameter, 8.80 base radius of plano power in Menicon 72 lens material, Nagoya, Japan), while the sBCL-only group received only OmniLenz. Bilateral dAM treatment was possible due to the 6 mm diameter central window in the amniotic membrane, which reduced the impact on the participant’s vision. All participants received a sBCL to maximise participant masking.

**Figure 2:**
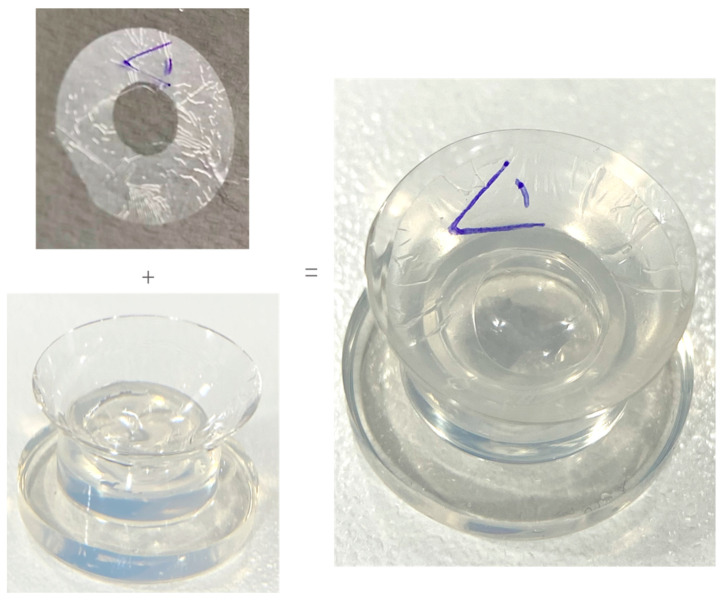
Loading of dAM onto sBCL.

Prior to application, the ocular surface was anesthetised (proxymetacaine hydrochloride 0.5%—Bausch & Lomb), and sensation was tested on the peripheral cornea with a surgical spear. Once the participant had no reaction to the stimuli, a specific BCL application was prepared. For participants receiving dAM+sBCL, Omnigen was loaded on the inner surface of the OmniLenz sBCL in an orientation such that the amniotic membrane’s epithelium was adjacent to the ocular surface, as per manufacturers recommendations (Figure 2). For participants receiving sBCL-alone, an imitation loading procedure emulating Omnigen loading was performed to aid participant masking. The prepared sBCL (with or without dAM) was then centrally applied to the cornea of each eye.

**Figure 3:**
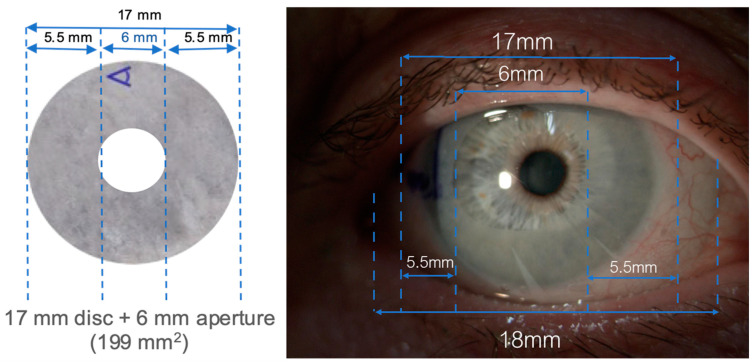
dAM + sBCL sizing on the ocular surface.

**Figure 4:**
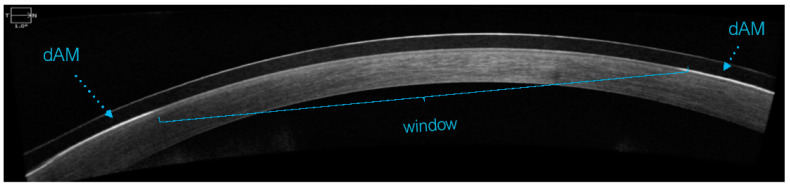
dAM centration check with OCT.

The sBCL fitting was assessed for dAM centration with a digital slit lamp (CSO Phoenix, Firenze, Italy) under 16× magnification (Figure 3) and an optical coherence tomographer (Cirrus-HD OCT, Zeiss Group, Oberkochen, Germany) (Figure 4).

The beneficial effects of the amniotic membrane typically last 3–9 days, over which time the membrane can either dissolve or become cloudy due to inflammatory coagulum accumulation [37,38,39,40]. Therefore, to allow a maximum dAM delivery while avoiding cloudiness, two consecutive bilateral applications of 4–5 days were used. Removal involved anaesthetising the ocular surface (proxymetacaine hydrochloride 0.5%—Bausch & Lomb) before removing and disposing of both the sBCL and dAM.

### 2.4. Measurements

Measurements followed TFOS DEWS II recommendations and were conducted in an ascending order of invasiveness [2,41]. Symptomatology was assessed with OSDI, Dry Eye Questionnaire 5-item (DEQ-5) and Symptoms Analysis iN Dry Eye (SANDE), frequency and severity visual analogue scales, at baseline/treatment day (day 0), and at all follow-up visits. Tear meniscus height (TMH), NIKBUT, lipid layer thickness (LLT), ocular hyperaemia, corneal (CornS) and conjunctival (ConjS) staining, lid wiper epitheliopathy staining (LWE), and in vivo confocal microscopy (IVCM) imaging of corneal nerve fibres and corneal dendritic cells (CDC) were assessed at baseline/treatment (day 0) and 1- and 3-month post-treatment follow-ups. Participants were assessed across all visits by a single clinician at the same location in a controlled environment (mean room temperature 21.5 ± 1.5 °C, relative humidity 43.2 ± 11.6%). Participants spent a minimum of 15 min acclimatising to the room conditions before being tested [41].

Symptomatology questionnaires OSDI, DEQ-5, and SANDE were used as recommended by their respective developers [42,43,44]. Ocular surface sign assessments were captured using the Oculus Keratograph 5M (Oculus, Wetzlar, Germany). TMH was assessed using infrared light and high-magnification digital imaging, and the averaged result was calculated from three measurements on the lower lid edge taken below the iris with calibrated digital callipers. NIKBUT was recorded as the time taken for the Placido disc reflections to show a first >5% distortion, while the participant-maintained fixation actively refraining from blinking; an average of 3 readings was recorded after two non-forceful blinks. LLT grading was evaluated by tear film interferometry on the modified Guillon-Keeler grading system: 0 (non-visible/absent), 1 (open meshwork), 2 (closed meshwork), 3 (wave/flow), 4 (amorphous), or 5 (coloured fringes) [45]. Bulbar and limbal (nasal and temporal regions) conjunctival hyperaemia was assessed in an automated and objective manner under high magnification on the JENVIS grading scale, to 0.1 precision [46]. Corneal staining (CornS) was assessed by wetting a fluorescein strip with saline (BioFluoro. Biotech Vision Care Pvt., Ahmedabad, India), shaking off the excess, and instilling it in the ocular outer canthus; a blue light and yellow observation filter were used for imaging [47]. Conjunctival staining (ConjS) was assessed by wetting a Lissamine strip (GreenGlo, Omni Lens Pvt., Ahmedabad, India) with a single drop of saline solution, keeping the drop on the strip for 5 s to elute the dye, and instilling the drop in the ocular outer canthus. The number of corneal and conjunctival punctate stained spots was subjectively graded and recorded as per the modified Oxford grading scheme (grades 0 to 5) [48]. The LWE staining assessment was subjectively graded and recorded relative to Korb’s grading scale [49]. Visual acuity was assessed in all visits for safety with an Early Treatment Diabetic Retinopathy Study LogMAR chart.

Corneal nerve branch density (CNBD no./mm^2^), fibre density (CNFD no./mm^2^), fibre length (CNFL mm/mm^2^), and total branch density (CNBD mm/mm^2^) were imaged using an IVCM (HRT3 with Rostock Corneal Module (RCM)—Heidelberg Engineering GmbH, Heidelberg, Germany) [50]. Scans were taken by applying a sterile polymethylmethacrylate cap (Tomo-Cap; Heidelberg Engineering GmbH, Heidelberg, Germany), filled with carbomer gel (Viscotears Liquid Gel. Bausch & Lomb, Kingston-upon-Thames, UK). The participants had 1 drop of proxymetacaine hydrochloride instilled in both eyes. Carbomer gel was placed on the Tomo-Cap to improve light transmission and optical coupling.

The central cornea was assessed by taking 3 clear sequence scans focused on the sub-basal nerve plexus (around 50–80 μm) without motion folds. The scan with the most nerves was assessed at every timepoint. The IVCM scanned a 400 × 400 μm corneal section with a microscope objective immersive lens of 63×. Calibration to mm^2^ was made by multiplying the CDCs in the frame by 6.25.

The corneal nerve data was analysed using the automated software ACCMetrics version 2.0, 03-2013 (University of Manchester, UK) [51]. Bright dendriform hyperreflective cell bodies were identified as immune corneal dendritic cells (CDCs), which were either mature (activated) or immature (non-activated), and their density (within each 1 mm^2^ area) was manually counted by a masked examiner.

### 2.5. Responder Analysis

The proportion of responders to treatment was calculated by assessing the change in measured parameters at 1- and 3-month timepoints compared to baseline. Responder analysis was conducted for corneal nerves and both mature and immature CDCs. Changes from baseline were categorised into three responder statuses: positive responder, negative responder, or non-responder. Responder statuses for overall (combined) nerve function were derived by aggregating individual parameter statuses.

No validated thresholds have been established for a clinically meaningful change in corneal nerve and CDC parameters. In the absence of published biomarker benchmarks for these parameters, except for normative values of corneal nerves [52], a standard 50% threshold change for CDC and 30% for corneal nerves was adopted to define a responder to treatment. This decision is supported by principles in clinical research that account for natural variability in measurements [52,53] and consider the observed change with contact lens wear, which is approximately 30% [54].

### 2.6. Statistical Analysis

Statistical analyses were performed using IBM SPSS Statistics version 26 (New York, NY, USA). Where data did not significantly differ from a normal distribution (Kolmogorov–Smirnov test, *p* > 0.05), repeated measure analysis of variance (ANOVA) was used initially, with paired sample *t*-test post hoc testing applied where overall significance was identified. All tests were two-tailed, and *p* < 0.05 was considered significant.

For the analysis of responder data, the Z-test was chosen for its appropriateness in comparing proportional differences. Care was taken to adjust for multiple comparisons where necessary, ensuring the robustness of our findings against type I errors. The chi-squared test was used to compare the independence of responder categories between the two treatments at each timepoint. These analyses were chosen based on the non-parametric nature of the data and the specific research questions regarding treatment efficacy over time.

## 3. Results

All participants enrolled had been diagnosed with DED and had been suffering with this condition for 12.5 ± 4.5 years. 81% of the participants were unsuccessfully managing their dry eye with different therapies, and 19% were not on current treatment. Some of the reported treatments were: 81% artificial tears (AT), 48% gel/ointments, 24% warm compress, 38% lid hygiene, 5% intense pulsed light (IPL), 5% steroids, and 5% antibiotics. In the management of refractory DED, most participants used some of the previously mentioned therapies in conjunction. The participant’s therapies were not changed during the six weeks prior to study enrolment.

Ninety-three participants were eligible and allocated for randomisation. On treatment day, intervention was given to the respective sBCL-alone (*n* = 39) and dAM+sBCL (*n* = 40) groups. Four participants were lost in the sBCL-alone group (two lost-to-follow-up and two discontinued), and five participants discontinued on the dAM+sBCL group (Figure 1).

A total of 70 participants (38 females and 19 males) with a mean ± SD age of 50.7 ± 18.1 completed the study. Allocation to treatment was as follows; dAM+sBCL (*n* = 35, aged 50.0 ± 16.8, 11 male) and BCL-alone (*n* = 35, aged 51.6 ± 19.4, 8 male) (Table 1). In each group, 71% and 11% of participants had severe (OSDI score 33–80) and very severe (OSDI > 80) disease [44], respectively, and 17% had moderate disease (OSDI score 23–32). The demographic characteristics of the participants are presented in Table 1. Most baseline characteristics did not differ between treatment groups, apart from LLT and CDC-activated (Table 1).

### 3.1. Symptomatology

**Figure 5:**
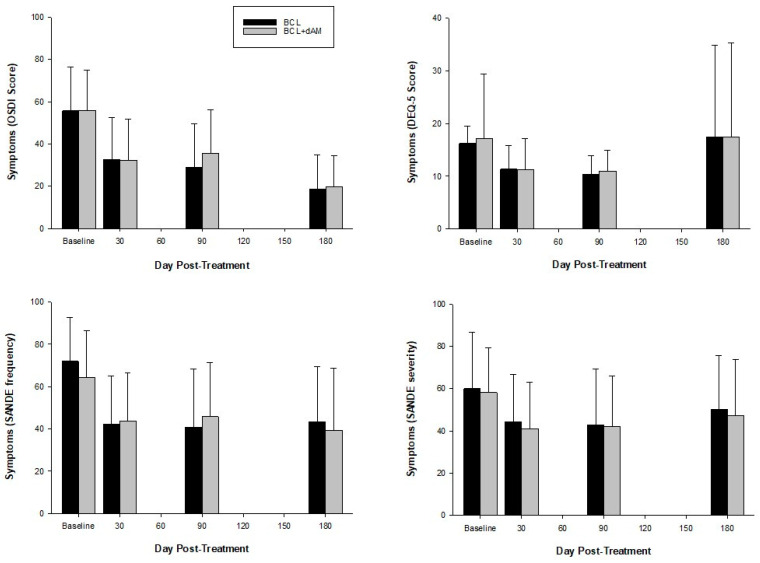
Symptomatology questionnaires pre- and post-treatment.

Symptoms reduced significantly with treatment (OSDI: F = 55,276, *p* < 0.001; DEQ-5: F = 4.579, *p* < 0.001; SANDE frequency: F = 19.716, *p* < 0.001 [Figure 5]; SANDE severity: F = 9.273, *p* < 0.001). The average baseline OSDI score (56.7 ± 18.5, Table 1) decreased by 40% and 65% by 1 and 6 months, respectively, which is effectively a transition from a severe to a mild disease stage, sustained for at least 6 months. Improvement was similar between groups (OSDI: F = 0.548, *p* = 0.462; DEQ-5: F = 0.140, *p* = 0.711; SANDE frequency: F = 0.131, *p* = 0.719; SANDE severity: F = 0.684, *p* = 0.413).

At 1 month, the proportion of participants positively responding to treatment compared to negatively responding, with a clinically meaningful improvement in OSDI score, was higher in the dAM-sBCL group (86%; *p* < 0.001) compared to the sBCL-alone group (82%; *p* < 0.001). At 3 months, there were 10% more positive responders in the dAM-sBCL treatment group compared to sBCL-alone (*p* = 0.673), with 97% of participants demonstrating a positive response and no negative responders. In the sBCL-alone group, more participants demonstrated a negative response than in the dAM-BCL group (10% vs. 6%). At 6 months, there was no statistical difference between treatment groups, and there was no negative responder in either group.

### 3.2. Signs

**Figure 6:**
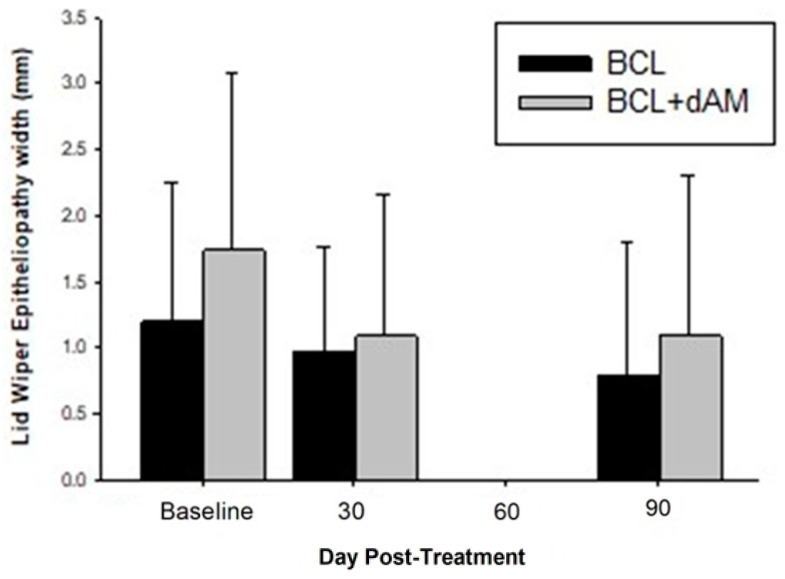
Lid Wiper Epitheliopathy pre- and post-treatment.

Corneal staining (CornS) (F = 3.419, *p* = 0.018) and conjunctival staining (ConjS) (F = 10.892, *p* < 0.001) were significantly reduced with both treatments but were similar between groups (F = 0.125, *p* = 0.724; F = 0.001, *p* = 0.972). Lid wiper epitheliopathy staining (LWE) did not reduce with treatment (F = 2.279, *p* = 0.081) and was similar between treatment groups (F = 1.126, *p* = 0.292), but decreased in width (F = 4.358, *p* = 0.005 (Figure 6)) following treatment, with no difference between treatment groups (F = 1.704, *p* = 0.198).

The change in ocular hyperaemia from baseline for limbal hyperaemia in the nasal region was a significant 0.35 ± 0.18 units lower with the dAM-sBCL compared to the sBCL-alone (*p* = 0.048). The change was higher in the bulbar than the limbal region (F = 180.589, *p* < 0.001), but was similar nasally to temporally (F = 2.510, *p* = 0.118) and did not change with time (F = 0.650, *p* = 0.584) or between treatment groups (F = 2.117, *p* = 0.150).

NIKBUT, TMH, and lipid thickness did not change significantly with time (F = 1.962, *p* = 0.145; F = 0.031, *p* = 0.993; F = 1.284, *p* = 0.281) or between treatment groups (F = 0.035, *p* = 0.853; F = 0.084, *p* = 0.773; F = 0.001, *p* = 0.999).

### 3.3. In Vivo Corneal Microscopy (IVCM) Analyses

#### 3.3.1. Corneal Nerves

Repeated measures ANOVA showed no significant change over time for either treatment group (Table 2).

The dAM-sBCL group exhibited a close to significant 15% improvement at 1 month for CNFD (t = −1.882, *p* = 0.070) (Table 3). Although the effect decreased slightly by 3 months, it remained 11% higher than baseline (t = −1.223, *p* = 0.231), indicating a potentially lasting positive trend. For CTBD, a marginally significant difference (t = −1.827, *p* = 0.043) between treatments at 1 month favoured the dAM-sBCL treatment, which exhibited a 13% increase from baseline. Collectively, this data suggests the rapid, but in some cases brief, benefit of dAM-sBCL over sBCL-alone, which decreases over time, explaining why repeated measures ANOVA did not demonstrate a significant treatment effect over time (Table 3).

The dAM-sBCL group showed no negative changes across most nerve parameters over time, with the exception of CNBD and CTBD at 3 months (Table 3). In contrast, sBCL-alone treatment exhibited a negative trend (ranging from 0.8% to 9% decrease) across all nerve parameters from baseline to 1 month; sBCL-alone treatment caused a negative impact trend to CNFD with a decrease from baseline to 1 month (5.2%, t = −0.681, *p* = 0.526), and in CNBD with a decrease from baseline to 1 month (9%, t = 0.598, *p* = 0.554). These parameters recovered by 3 months, (12.2%, t = −1.219, *p* = 0.232, and 14%, t = −0.769, *p* = 0.448, respectively), though the changes were not statistically significant. This phenomenon also explained the marginally significant improvement between 1 and 3 months for both CNFD (t = −1.864, *p* = 0.072) and CNFL (t = −1.906, *p* = 0.066). Any negative effect in the remaining sBCL-treatment group nerve parameters was also reversed by 3 months from baseline, with no significant differences between treatments (Table 3).

Responder analysis (based on the number of participants demonstrating 30% improvement in nerve parameters) of the combined nerve response and individual nerve parameters corroborated the clinical assessment (Table 4). At 1 month, the dAM-sBCL treatment group demonstrated a significantly higher proportion of positive responders (74%) compared to the sBCL-alone group (29%) (s = 3.87, *p* < 0.001) in the combined nerve parameter. This indicates that participants treated with dAM-cBCL experienced a more immediate improvement in corneal nerve health. In contrast, sBCL-alone treatment showed a higher proportion of negative responders (65% vs. 14% for dAM+sBCL), indicating an early negative impact on nerve health in DED patients (s = 4.32, *p* < 0.001), when used without dAM, suggesting a stand-alone BCL treatment may negatively impact nerve health in DED patients.

By 3 months, the positive responder rate between groups for the combined nerve parameter was not statistically significant (s = 0.16, *p* = 0.870). However, it is notable that within the dAM-sBCL treatment group, the rate of positive responders was significantly higher at both 1 (s = 3.87, *p* < 0.001) and 3 months (s = 4.54, *p* < 0.001), highlighting its sustained benefit compared to sBCL-alone. In the sBCL-alone group, negative responders were significantly higher at 1-month (s = 2.09, *p* = 0.036).

Breaking down the combined nerve responder data into individual corneal nerve parameters, the proportion of positive responders compared to negative responders was consistently higher with dAM-sBCL at both time points (Table 4). This significant effect continued for 3 months for CNFL (s = 3.3, *p* = 0.001), and CNFD (s = 2.56, *p* = 0.011).

Overall, dAM+sBCL significantly increased positive nerve responder rates (*p* < 0.001), while sBCL-alone appeared to have a significant negative impact on nerve health responder rates (*p* < 0.001).

#### 3.3.2. Corneal Dendritic Cells (CDCs)

##### Mature CDCs

There was a statistically significant difference between the sBCL-alone and dAM-BCL treatments at baseline (*p* = 0.005) for activated CDCs, with a higher value in the dAM-sBCL group (Table 1). Therefore, the difference from baseline at 1 (*p* = 0.185) and 3 months (*p* = 0.540) was analysed, but it was not significant. The differences between treatments are not statistically significant at 1 and 3 months, with *p*-values of 0.247 and 0.426, respectively.

There was a trend towards significance in temporal effect (F(2,62) = 2.7663, *p* = 0.070) in the dAM-sBCL treatment group (F(2,62) = 2.7663, *p* = 0.070), but not in the sBCL-alone group (*p* = 0.740). There was a notable reduction in mature CDCs in the dAM-sBCL treatment group from baseline to both 1 (22%, t = 1.843, *p* = 0.056) and 3 (30%, t = 2.342, *p* = 0.025) months. The difference between treatments at 3 months was also approaching significance in favour of dAM-sBCL (*p* = 0.068). This data indicates dAM-sBCL has a greater effect on reducing mature CDC than the sBCL-alone treatment and that this effect remained relatively stable through 3 months. The mixed-effects model indicated no significant interaction between time and treatment (*p* > 0.05).

##### Immature CDC

Analysis of immature CDCs post-treatment showed no significant temporal effect in the sBCL-alone treatment group (F(2,68) = 2.1106, *p* = 0.129). Conversely, the dAM-sBCL group demonstrated a significant effect (F = 5.355, *p* = 0.006). The mixed-effects model revealed no significant interaction between time and treatment (*p* > 0.05) (Table 2).

Both treatment groups increased immature CDCs at 1 month, but only sBCL+dAM was significant at 55% (T = 2.4972, *p* < 0.01). This was followed by a non-significant 20.1% decrease from 1 month to 3 months (T = −1.3405, *p* = 0.185) (Table 4). Neither group showed a statistically significant treatment effect over time (repeated measures), though dAM+sBCL was close to significance (F(2,64) = 2.6348, *p* = 0.079, Table 2).

**Figure 7:**
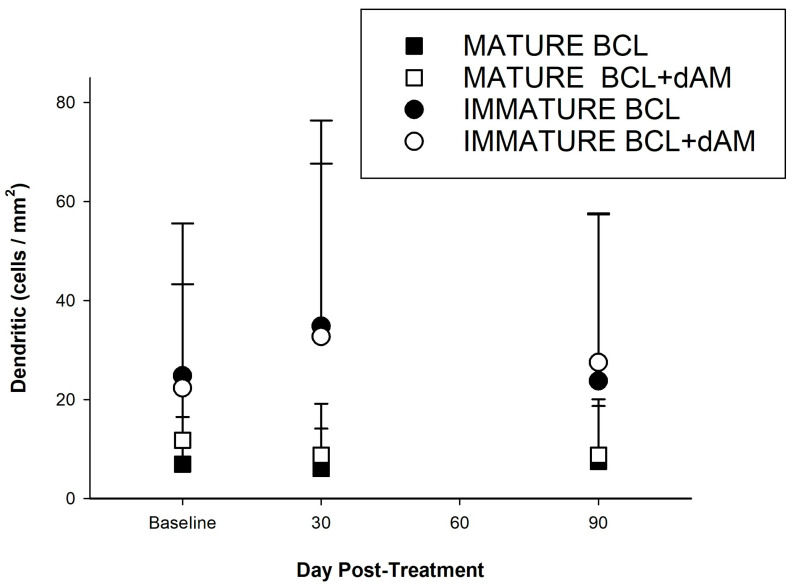
Corneal dendritic cell numbers (cells/mm^2^) pre- and post-treatment.

These results suggest that while the dAM-sBCL treatment may show a significant increase in immature cells at 1 month, the effect is not sustained or further improves significantly at 3 months (Figure 7). Conversely, the sBCL-alone treatment does not demonstrate significant changes at either time point in this analysis.

Responder analysis showed that there were more participants with a reduction in mature CDC count (31%, s = 1.73, *p* = 0.083) and an increase in immature CDC count (55%, s = −3.07, *p* = 0.002)) in the dAM-sBCL group, not generally observed with the sBCL alone (Table 5). Conversely, sBCL-alone treatment resulted in more participants with the opposite responder status for mature and immature CDC at both time points (Table 6).

### 3.4. Safety

Repeated measures ANOVA indicated that there were no significant changes over time within either the sBCL-alone or the dAM-sBCL treatment groups. However, the dAM+sBCL treatment group showed a trend towards significance (*p* = 0.091), indicating potential benefits over time.

The safety profile of the treatment was good, in this study, although 63% of eyes experienced a level of lens-related discomfort during the insertion period of wear time, with dryness/grittiness (56%), blurred vision (50%), irritation/soreness (18%), redness/swelling (13%), pain (4%), and photophobia (3%) and headaches (3%), being the reported causative symptoms. No serious adverse events were reported.

## 4. Discussion

This prospective randomised controlled trial provides level 1 clinical evidence for the long-term efficacy of sutureless dehydrated amniotic membrane (dAM). This is the first RCT, known to the authors, to have a dAM delivered to the ocular surface (by a specialised bandage contact lens applied bilaterally, allowing reasonable vision while having the treatment delivered), compared to the specialised soft bandage contact lens on its own, for the management of moderate-to-severe dry eye disease.

Participant symptomatology was seen to decrease by around 65% at 6 months. The data also supports the long-term efficacy of the treatment in reducing disease severity and improving patient outcomes, remaining stable for at least 6 months, with the highest benefit happening at 1 month in the dAM+sBCL group. Not only did the OSDI scores decrease, but also the severity and frequency of the self-reported symptomatology. The initial month showed a significant reduction in symptoms for a large majority of participants, 88% in the dAM+sBCL vs. 78% in the sBCL-alone, with a slightly reduced but still notable effectiveness in over 65% and 55% of participants at six months, respectively. Compared to the lens group, the dAM treatment demonstrated superior efficacy in managing symptom severity, both initially and over a longer period of time. This symptomatology improvement went hand-in-hand with ocular surface improvements, which is consistent with previously published data [28,55,56,57].

Even though a previous RCT using cryopreserved AM only treated one eye [56], this study uniquely involved treatment of both eyes and only analysed the right one in order to avoid possible different symptomatology scores between eyes.

The baseline level of corneal staining in this study cohort was relatively low, with 23% and 44% of participants having no or grade 1 staining at baseline, respectively. Overall, the participant cohort had an average baseline grade of just 1.37 on the Oxford scale (0–5). This is because DED can be diagnosed by a positive homeostasis maker, either by ocular surface staining, a NIKBUT lower than 10 s, high osmolarity, or osmolarity differences, all together with a positive questionnaire for symptomatology [2]. In this study, most DED participants were diagnosed with a low NIKBUT. Consequently, the response rates to the treatment for corneal staining were modest, with a marginally statistically significant 36% of participants showing improvement at one month, increasing slightly to 42% at three months. Conjunctival staining, although somewhat higher than corneal staining, was also low at baseline, with an average grade of 1.80 on the Oxford Scale. The mechanical stress between the lid and ocular surface, observed in the form of LWE, did decrease in width after treatment use, but LWE was similar between treatments.

NIKBUT, TMH, and LLT showed no statistically significant change with treatment, and the response was similar between treatment arms. An RCT found that restoration of the ocular surface requires prolonged and consistent treatment use in order for clinical sign changes to be observed [58]. A change in LWE was reported from day 60 post-treatment, LLT from day 90, NIKBUT, and staining from day 120; this could be a reason why this study found no significant changes in staining but did in LWE within the study’s assessment timeframe.

Analysis of the various corneal nerve parameters at individual timepoints also demonstrated the potential benefit of dAM-sBCL treatment for DED compared to the use of sBCL-alone. After three months, more than half of the treated patients continued to benefit from the treatment across all nerve health parameters, even though the overall difference between groups was not statistically significant. After three months, almost 75% of participants continued to benefit from the treatment across their combined nerve health. Overall, the proportion of positive responders following dAM+sBCL treatment did not decline (combined nerve health), indicating stability in the responder rate to dAM over time.

The dAM-sBCL treatment seems to offer a strong and stable treatment effect from the start on CNFL, making it a potentially better choice for rapid improvement. While treatment with sBCL alone caused an initial negative impact, the effect appears to progressively recover.

Corneal nerve density after dAM-sBCL treatment appears to be more effective initially (or sBCL-alone more detrimental), with a higher proportion of positive responders at the 1-month mark. Similar findings were reported in another study using cryopreserved dAM, with an improvement in nerve density at 1 month of approximately 4 μm/mm^2^ [56]. This study found that this advantage normalises over time, aligning more closely with BCL by the 3-month mark, but this is contradictory to the 3-month findings reported by other authors [56], where the nerve density still increased by 2.5 μm/mm^2^ from the 30-day visit to the 90-day visit [56]. In our study, the stability in responder rates from 1 to 3 months suggests that initial responses are likely to persist, which is also reported in previous literature [56].

Post-dAM-sBCL treatment, corneal branch density shows a stronger initial positive response, which is advantageous for early treatment outcomes. sBCL-alone, levelled up by the 3-month mark, indicating an opportunity to explore optimising the dAM-sBCL treatment protocol. The initial negative effect on corneal nerves of the sBCL-alone treatment recovered such that there was no statistically significant difference between treatment groups at 3 months. Both treatments maintain their initial responder rates over time, which is beneficial for predicting long-term outcomes based on early treatment responses. The data suggests that dAM-sBCL delivers early efficacy with a high rate of positive responders at the 1-month mark compared to sBCL-alone, which has also been proven in a previous RCT at the same time-point [56].

The data from this RCT indicates a robust and rapid initial response to treatment in improving all aspects of corneal nerve health. While there is a slight decrease in responders over time, the overall benefit of the dAM-sBCL treatment remains compared to sBCL. This highlights the protective and therapeutic benefits of dAM-sBCL in enhancing corneal nerve health, while mitigating the negative impacts observed with sBCL alone. The positive trends in several nerve parameters with dAM-sBCL treatment, even if not always statistically significant, indicate potential benefits warranting further investigation.

Importantly, the dAM-sBCL group consistently showed no negative corneal nerve changes across the remaining measured parameters, and while not all nerve parameters were significantly improved, this indicated dAM-sBCL does not negatively impact nerve health. This is an important safety consideration for the long-term use of dAM-sBCL in DED treatment. Conversely, the sBCL-alone group had a higher proportion of negative responders at 1 month, which, although reversed at 3 months, still trended higher compared to dAM-sBCL. Even if the use of BCLs is adequate for shielding and protecting the ocular surface in certain ocular surface conditions, benefiting patients on pain control and ocular surface measures [59,60], this study noticed that corneal nerve findings suggested that the routine use of sBCL-alone, without dAM, in DED patients may compromise nerve health in the short term and can induce a sub-clinical inflammatory response showcased by the presence of CDCs, which was also noted in other studies [54,61]. Given that healthcare professionals cannot routinely assess corneal nerves, there is a risk of unseen, long-term damage. Therefore, the potential risks of using sBCL alone should be carefully considered.

The corneal inflammatory status can be assessed through the inflammatory cells present on the corneal plexus by counting the number of CDC present. CDCs are antigen-specific cells that become activated or migrate [62] to the challenged structure in the presence of an inflammatory [63] or infectious state [64], such as contact lens wear [64,65,66], dry eye [11,67] or keratitis [54]. CDCs play an important role in the innate and adaptive arms of the immune system in the ocular surface immune response by modulating inflammation. In the context of DED, the activation state of CDCs, categorised as either immature (non-activated) or mature (activated), can significantly influence disease progression and symptom severity [63]. CDCs typically transition from an immature/inactivated state to a mature/activated state in response to inflammatory stimuli; this is an early and sensitive measure of an imminent inflammatory process [54,68,69]. Immature CDCs are primarily involved in antigen capture and processing, whereas mature CDCs are responsible for antigen presentation and the initiation of adaptive immune responses. Both mature and immature CDCs are known to be increased compared to controls in DED [11]. Therefore, it is thought that the numbers of immature and mature CDCs can reflect different stages of dendritic cell activation and maturation in response to ocular surface inflammation. Even uncomplicated contact lens wear has been reported to lead to an immediate subclinical inflammatory response. This response has been characterised by an increase in CDCs during contact lens wear and has been previously reported to happen with a peak at 2 h of wear [54]. In our study, CDCs were measured at 1 and 3 months, but this could have happened during the 1-week treatment time.

In terms of mature CDCs, no significant change from baseline at both 1-month and 3 months was observed following sBCL-alone treatment, suggesting that the effects observed at 1-month remained relatively stable through 3 months. The dAM+sBCL group has shown a greater reduction in activated CDCs over time, with a 30% decrease being significant at 3 months post-baseline. These results suggest that while there may be initial differences in the conditions of the groups at baseline (Figure 1), the treatments do differ significantly in their effects, specifically at 3 months (Table 5).

For the immature CDCs, dAM+sBCL demonstrates a significant increase in cells at 1 month but then decreases over 3 months. Very similar findings were reported by John et al. on their RCT with cryopreserved AM. The undefined (immature) CDCs increased at 1 month, followed by a decrease at 3 months, both not being statistically significant [56].

This observed increase in immature (inactive) CDCs in the dAM+sBCL treatment group suggests that, as a result of an immunomodulatory/anti-inflammatory effect, dAM+sBCL likely prevents the activation and maturation of CDCs. This is consistent with an anti-inflammatory mechanism where immature CDCs accumulate as their activation is curtailed, suggesting that dAM+sBCL is effective in halting the progression of immature CDCs to their mature state, thereby exerting the desired anti-inflammatory effect. This anti-inflammatory effect may lead to improved clinical outcomes for patients with DED by modulating the immune response on the ocular surface. By preventing or modulating CDC activation, dAM+sBCL may help manage DED, which is characterised by chronic inflammation, but it also suggests multiple potential applications in other ocular surface inflammatory conditions while promoting ocular surface health.

Early differences in treatment responses suggest that patient selection for dAM-sBCL could be strategised based on the speed of response required. The consistent effectiveness of dAM-sBCL makes it suitable for situations where immediate improvement is critical, such as persistent/recurrent epithelial defects [29,70,71,72], non-healing corneal ulcerations, microbial keratitis [73], neurotrophic keratitis [74], chemical/thermal burns [37,75], limbal stem cell deficiency [76,77], DED [28,33,57], ocular neuropathic pain [78], Steven’s Johnson syndrome [79], graft vs. host [27], any cicatrising eye disease, or situation when inflammation, angiogenesis, and fibrosis need to be controlled.

These findings can help guide clinical decisions, especially when choosing between sBCL and dAM-sBCL for treating DED related to corneal nerve damage or abnormalities, specifically, dAM-sBCL may be preferable for quicker results. Further studies could help clarify the long-term effects and potential for changes in treatment efficacy over time beyond the 3-month period.

Although previous studies performed similar treatments using amniotic membranes (cryopreserved) [28,57,59], only one other RCT looked into the corneal nerve status pre- and post-cryopreserved amniotic membrane treatment [56]. Even though their study performed a single application over 3–5 days, compared to 8–10 days in this study, the results are comparable.

A key difference between our study and the RCT published by John et al. [56], is the use of sBCL as the control arm, whereas the control group in John’s study was the participant’s normal dry eye therapy. BCLs are recognised as a treatment option for corneal exposure and surface healing and are listed in step 3 of the Tear Film and Ocular Surface Society (TFOS) Dry Eye Workshop (DEWS) staged management approach for DED [18]. Given the potential therapeutic benefits of BCLs, it is not surprising that differentiating the symptomatology effects between dAM-sBCL and sBCL alone presented a significant challenge. This underscores the importance of the benefits presented by dAM-sBCL over sBCL in nerve health and immune cell parameters.

The conclusions of this RCT could have also been limited by; the potential misidentification of elements in the IVCM image as CDCs cells and morphological cell change, especially during inflammatory activation of CDCs (enlargement of dendrites might occur during lens wear) [61]. In addition, the relatively low baseline levels of ocular surface staining in both corneal and conjunctival areas might have contributed to the modest response rates observed post-treatment. Furthermore, the lack of evidence on dAM retention and dissolution while under the lens made tracking the treatment duration and activity of treatment on the eye more difficult to monitor.

Further research with daily follow-ups to assert continuity of treatment on the eye in combination with extended follow-up periods is necessary to understand the long-term effects of dAM+sBCL treatment on ocular signs, symptoms, and CDC dynamics. Understanding the clinical and economic benefits of central window and bilateral treatment will be important for mainstream adoption, while investigating the molecular mechanisms by which dAM+sBCL inhibits CDCs maturation could provide valuable insights into its anti-inflammatory properties and broader therapeutic potential.

## 5. Conclusions

This study has demonstrated that an 8–10-day application of dAM under a sBCL provides a long-term reduction in symptomatology for patients with DED. The reduction of 40% by 1 month and 65% by 6 months will have a marked impact on patients’ quality of life. There are also additional ocular surface benefits. By preserving and protecting nerve health and modulating the activation of CDCs, dAMs contribute to improved ocular surface health and reduce inflammation, both on an immediate and sustained long-term basis. This study supports the use of dAM-sBCL as a promising treatment strategy for managing moderate-to-severe DED effectively and safely.

## Figures and Tables

**Table 1:** Baseline demographic characteristics of participants randomised to sBCL-alone and dAM+sBCL. *n* = 70. Data are presented as mean ± standard deviation.

Characteristic	BCL-Alone	dAM+sBCL	*p* Value
Age	50.820 ± 19.14	49.76 ± 17.0	0.813
Gender	8 males, 27 females	11 males, 21 females	0.373
OSDI	55.71 ± 20.67	56.71 ± 18.53	0.831
DEQ-5	16.24 ± 3.32	14.81 ± 2.58	0.123
SANDE Frequency	71.92 ± 20.85	65.04 ± 22.68	0.312
SANDE severity	59.96 ± 26.66	59.67 ± 19.77	0.959
VA (LogMAR)	0.08 ± 0.17	0.12 ± 0.15	0.473
TMH (mm)	0.30 ± 0.13	0.26 ± 0.09	0.125
Hyperaemia Bulbar temporal (grade)	1.16 ± 0.53	1.11 ± 0.45	0.629
Hyperaemia Bulbar nasal (grade)	0.95 ± 0.46	1.09 ± 0.51	0.242
Hyperaemia Limbal temporal (grade)	0.82 ± 0.41	0.79 ± 0.36	0.772
Hyperaemia Limbal nasal(grade)	0.74 ± 0.42	0.79 ± 0.40	0.545
NIKBUT (seconds)	8.33 ± 5.91	7.84 ± 4.66	0.710
LLT (grade)	3.20 ± 1.61	4.14 ± 1.14	0.007 *
CornS (grade)	1.14 ± 1.35	1.37 ± 1.21	0.469
ConjS (grade)	1.63 ± 0.94	1.80 ± 1.28	0.841
LWE Length (grade)	1.51 ± 1.48	1.97 ± 1.40	0.133
LWE Width (grade)	1.20 ± 1.05	1.74 ± 1.34	0.060
CNFD (no./mm^2^)	20.36 ± 10.88	23.16 ± 11.36	0.252
CNBD (no./mm^2^)	26.47 ± 25.24	33.64 ± 37.02	0.363
CNFL (mm/mm^2^)	13.63 ± 4.17	13.79 ± 5.82	0.659
CTBD (mm/mm^2^)	49.26 ± 35.74	54.78 ± 61.39	0.638
CDC Activated (no./mm^2^)	6.96 ± 9.55	11.78 ± 10.03	0.005 *
CDC Inactivated (no./mm^2^)	24.82 ± 30.75	22.32 ± 20.97	0.672

CDC: corneal dendritic cell, CNBD: corneal branch density, CNFD: corneal nerve fibre density, CNFL: corneal nerve fibre length, CTBD: corneal total branch density, DEQ-5: dry eye questionnaire—5 item, LLT: lipid layer thickness, LWE: lid wiper epitheliopathy, NIKBUT: non-invasive keratometric break-up time, OSDI: ocular surface disease index, SANDE: symptoms analysis in dry eye, TMH: tear meniscus height, VA: visual acuity. * denotes statistical differences in between groups prior study treatment allocation.

**Table 2:** Comparison of corneal nerve parameter change with treatment and between treatments over time. *n* = 70.

Feature (Units)	With Treatment (Over Time)	Between Treatments
CNFD no./mm^2^	F = 1.072, *p* = 0.363	F = 2.005, *p* = 0.163
CNBD no./mm^2^	F = 0.178, *p* = 0.837	F = 1.810, *p* = 0.184
CNFL mm/mm^2^	F = 2.162, *p* = 0.120	F = 1.43-, *p* = 0.237
CTBD mm/mm^2^	F = 0.035, *p* = 0.966	F = 1.098, *p* = 0.299
CDCs activated no./mm^2^	F = 0.779, *p* = 0.461	F = 0.138, *p* = 0.711
CDCs deactivated no./mm^2^	F = 5.355, *p* = 0.006 *	F = 1.261, *p* = 0.266

CNFD: corneal nerve fibre density, CNBD: corneal branch density, CNFL: corneal nerve fibre length, CTBD: corneal total branch density. * denotes statistical significance.

**Table 3:** Individual nerve parameter timepoint analysis. *n* = 70.

Parameter	Baseline	Change— 1 Month	*p* Value	Change— 3 Months	*p* Value
CNFL					
sBCL-alone	14.52 ± 3.72	−0.8% (−0.11)	0.908	10% (+1.45)	0.013 *
dAM+sBCL	14.3 ± 3.43	9.7% (+1.4)	0.170	6.7% (0.82)	0.359
*p* value	0.927	0.379		0.466	
CNFD					
sBCL-alone	22.46 ± 5.11	−5.2% (−1.17)	0.526	12.2% (+2.7)	0.232
dAM+sBCL	19.17 ± 4.82	15.2% (+2.92)	0.070	10.9% (+2.1))	0.231
*p* value	0.253	0.791		0.190	
CNBD					
sBCL-alone	29.43 ± 4.12	−8.9% (−2.62)	0.554	14.4% (+4.23)	0.448
dAM+sBCL	32.71 ± 4.32	15.9% (+5.21)	0.501	−3.2% (−1.04)	0.869
*p* value	0.687	0.114		0.774	
CTBD					
sBCL-alone	47.26 ± 6.12	−3.3% (−1.56)	0.797	9.9% (+4.69)	0.504
dAM+sBCL	53.88 ± 6.32	12.4% (+6.68)	0.599	−7.6% (−4.09)	0.681
*p* value	0.620	0.043 *		0.817	

* denotes statistical significance.

**Table 4:** Responder analysis for corneal nerve outcome measures using a 30% response threshold. *n* = 70.

	1-Month 100% Participants (*n* = 35)			3-Month 100% Participants (*n* = 35)		
Combined Nerves	dAM-sBCL	sBCL-alone	dAM-sBCL vs. sBCL-alone	dAM-sBCL	sBCL-alone	dAM-sBCL vs. sBCL-alone
Responders	74% (26)	29% (10)	<0.001 *	74% (26)	74% (25)	0.870
Negative responders	14% (5)	65% (22)	<0.001 *	11% (4)	21% (7)	0.245
*p* value	<0.001 *	0.037 *		<0.001 *	<0.001 *	
CNFL	dAM-sBCL	sBCL-alone	dAM-sBCL vs. sBCL-alone	dAM-sBCL	sBCL-alone	dAM-sBC Lv sBCL-alone
Positive responders	34% (11)	15% (5)	0.072 *	41% (13)	18% (6)	0.424
Negative responders	9% (3)	9% (3)	0.658	13% (4)	6% (2)	0.610
*p* value	0.011 *	0.317		0.001 *	0.046 *	
CNFD	dAM-sBCL	sBCL-alone	dAM-sBCL vs. sBCL-alone	dAM-sBCL	sBCL-alone	dAM-sBCL vs. sBCL-alone
Positive responders	35% (11)	15% (5)	0.142	47% (15)	26% (9)	0.430
Negative responders	13% (4)	24% (8)	0.320	19% (6)	15% (6)	0.950
*p* value	0.006 *	0.239		0.011 *	0.131	
CNBD	dAM-sBCL	sBCL-alone	dAM-sBCL vs. sBCL-alone	dAM-sBCL	sBCL-alone	dAM-sBCL vs. sBCL-alone
Positive responders	56% (18)	33% (11)	0.018 *	42% (13)	45% (15)	0.610
Negative responders	34% (11)	42% (14)	0.675	32% (10)	27% (9)	0.290
*p* value	0.066	0.228		0.376	0.058	
CTBD	dAM-sBCL	sBCL-alone	dAM-sBCL vs. sBCL-alone	dAM-sBCL	sBCL-alone	dAM-sBCL vs. sBCL-alone
Positive responders	59% (19)	33% (11)	0.004 *	42% (13)	45% (15)	0.76
Negative responders	34% (11)	42% (14)	0.867	39% (12)	27% (9)	0.59
*p* value	0.039 *	0.206		0.777	0.564	

Significant differences were found in the distribution of response types between dAM+sBCL and sBCL-alone at 1 month, supporting the initial efficacy of dAM. * denotes statistical significance.

**Table 5:** CDC analysis. *n* = 70.

Treatment	% Change: 1 Month (Units)	*p* Value	% Change: 3 Month (Units)	*p* Value	Effect over Time
Mature CDCs					
sBCL-alone	−12.82% (−0.15)	0.922	7.69% (0.09)	0.563	0.740
dAM+sBCL	−22.22% (−0.44)	0.056	−30.16% (−0.59)	0.025 *	0.070
variance	−9.4% (−0.29)		−37.85% (−0.68)		
*p* value	0.184		0.068		
Immature CDCs					
sBCL-alone	41% (1.63)	0.248	−4% (−0.17)	0.656	−0.073
dAM+sBCL	54.6% (1.97)	0.009 *	34.5% (1.24)	0.153	0.079
variance	13% (0.34)		38.5% (1.41)		
*p* value	0.375		0.108		

* denotes statistical significance.

**Table 6:** Responder analysis for mature (activated) and immature (deactivated) CDCs—50% change threshold. *n* = 70.

	1-Month 100% Participants (*n* = 70)			3-Month 100% Participants (*n* = 70)		
Mature CDCs	dAM+sBCL	sBCL-alone	dAM-sBCL vs. sBCL-alone	dAM+sBCL	sBCL-alone	dAM-sBCL vs. sBCL-alone
Positive responders	31% (10)	25% (8)	0.929	47% (15)	16% (5)	0.384
Negative responders	25% (8)	50% (16)		25% (8)	56% (18)	
*p* value	0.083	0.447		0.055 *	0.792	
Immature CDCs	dAM+sBCL	sBCL-alone	dAM-sBCL vs. sBCL-alone	dAM+sBCL	sBCL-alone	dAM-sBCL vs. sBCL-alone
Positive responders	18% (6)	23% (8)	0.634	25% (8)	40% (14)	0.165
Negative responders	55% (18)	51% (18)		52% (17)	23% (8)	
*p* value	<0.002 *	0.013 *		0.022 *	0.122	

* denotes statistical significance.

## Data Availability

Data is unavailable due to privacy restrictions.
